# Age-specific modulation of intermuscular beta coherence during gait before and after experimentally induced fatigue

**DOI:** 10.1038/s41598-020-72839-1

**Published:** 2020-09-28

**Authors:** Paulo Cezar Rocha dos Santos, Claudine J. C. Lamoth, Fabio Augusto Barbieri, Inge Zijdewind, Lilian Teresa Bucken Gobbi, Tibor Hortobágyi

**Affiliations:** 1 Department of Human Movement Sciences, University Medical Center Groningen, University of Groningen, Groningen, The Netherlands; 2 Posture and Gait Studies Laboratory (LEPLO), Institute of Biosciences, Graduate Program in Movement Sciences, São Paulo State University (UNESP), Rio Claro, Brazil; 3 Department of Physical Education, Human Movement Research Laboratory (MOVI-LAB), Graduate Program in Movement Sciences, São Paulo State University (UNESP), Bauru, Brazil; 4 Department of Biomedical Sciences of Cells and Systems, University of Groningen, University Medical Center Groningen, Groningen, The Netherlands; 5 Present Address: Department of Human Movement Sciences, University of Groningen, University Medical Center Groningen, A. Deusinglaan 1, 9713 AV Groningen, The Netherlands

**Keywords:** Physiology, Neuroscience, Motor control, Neural ageing

## Abstract

We examined the effects of age on intermuscular beta-band (15–35 Hz) coherence during treadmill walking before and after experimentally induced fatigue. Older (n = 12) and younger (n = 12) adults walked on a treadmill at 1.2 m/s for 3 min before and after repetitive sit-to-stand, rSTS, to induce muscle fatigability. We measured stride outcomes and coherence from 100 steps in the dominant leg for the synergistic (biceps femoris (BF)-semitendinosus, rectus femoris (RF)-vastus lateralis (VL), gastrocnemius lateralis (GL)-Soleus (SL), tibialis anterior (TA)-peroneus longus (PL)) and for the antagonistic (RF-BF and TA-GL) muscle pairs at late swing and early stance. Older vs. younger adults had 43–62% lower GL-SL, RF-VL coherence in swing and TA-PL and RF-VL coherence in stance. After rSTS, RF-BF coherence in late swing decreased by ~ 20% and TA-PL increased by 16% independent of age (p = 0.02). Also, GL-SL coherence decreased by ~ 23% and increased by ~ 23% in younger and older, respectively. Age affects the oscillatory coupling between synergistic muscle pairs, delivered presumably via corticospinal tracts, during treadmill walking. Muscle fatigability elicits age-specific changes in the common fluctuations in muscle activity, which could be interpreted as a compensation for muscle fatigability to maintain gait performance.

## Introduction

Healthy aging modifies the integrated and coordinated neural control of gait^1,2^. The age-related modifications in neuromuscular control are evidenced, among others, by the 40–50% increases in rectus (RF)-biceps femoris (BF) coactivation at heel strike during gait^3–5^. These alterations in neuromuscular control during gait are usually associated with age-related reductions in mechanical output at the ankle and increases in mechanical work generation at the hip joint^6^. Age-specific decreases in inhibitory cortical control are accompanied by decreases in Ia afferent input^7^ that may lead to a depression of the inputs from muscle afferents compensated by increases in coactivation between agonist and antagonistic muscles during gait^3,4^. Such reductions in inhibitory cortical control impair cortico-muscular communication by reducing the strengths of inputs via the corticospinal tract during muscle contraction^8^, indirectly reflected by changes in muscular coherence during walking^1,9,10^.

Intermuscular coherence is a correlational measure signifying the strength in the frequency domain between two signals recorded from two muscles, suggesting the oscillatory synchronization of action potentials discharged by motor units^1,11^. This oscillatory synchronization is indirectly related to common presynaptic inputs to the motor neuron pool of two or more muscles^12–14^, as an attempt by the central nervous system (CNS) to reduce the dimensionality and complexity of control^15^. Data from neurological patients suggest that beta-band coherence in the range of 15–35 Hz emanates from cortical structures, including the sensorimotor cortex to control presynaptic inputs^12,16^. In older adults, there is diminished beta-band intramuscular coherence during swing phase, which may indicate a reduced common drive within the proximal and distal part of the tibialis anterior (TA) muscle^1^, results consistent with data in hand muscles^17^. However, how healthy aging affects coherence among muscles crossing the ankle, knee and hip joints during such a functional task as gait has not yet been examined. Intermuscular coherence could provide information on age-related neuromuscular adaptation by inferring the oscillatory coupling of muscles spanning lower extremity joints.

Age-typical reductions in neuromuscular function are associated with an increase in effort to execute activities of daily living^18^. The high effort-demand can increase susceptibility to muscle fatigability during gait^19^. Muscle fatigability, i.e., a decline in the capacity to generate voluntary force, affects motor control^20–23^. A consequence of exercise-induced fatigability is a decrease in the dimensionality of the control^24^ by increasing common input to several muscles^25–27^, which would imply impaired motor coordination during complex tasks^28^. Indeed, exercise-induced fatigability is likely to be related to changes in the excitability along the neural axis, which might increase the coupling of oscillatory neural inputs, reflecting in the common fluctuation in muscle activity across muscle pairs^25,26^. Indeed, experimental data suggest 50–90% increase in beta-band cortico-muscular and muscular-muscular coherence during voluntary contraction^28–30^ that may evolve as a neural compensatory strategy for the fatigue-induced decline in performance in healthy younger adults^23,26,28,29,31^. Because fatigue perturbs neuromuscular control, fatigability protocols have often been used to probe older adults’ gait adaptability in response to this form of internal perturbations^32–36^.

Repetitive sit-to-stand (rSTS) is a model to induce muscle fatigability in lower extremity muscles and probe gait adaptability^37^. Following bouts of rSTS, maximal voluntary force, the associated surface electromyographic (EMG) activity, and median frequency of muscles involved in gait can decrease up to 18%^35,38^. However, previous studies found only weak evidence for age-related adaptations in stride metrics after fatigue^35^. The possibility exists that age-specific neural compensations to fatigue would minimize deterioration in stride metrics and preserve gait performance. In the present paper, we explore this hypothesis by further probing adaptive neural mechanisms through intermuscular coherence in the beta-band. Therefore, we determined the effects of age on beta-band coherence during treadmill walking before and after experimentally induced fatigability. Based on age-typical distal to proximal reductions in mechanical work generation during treadmill walking^6^, we hypothesized that older compared with young adults would exhibit lower coherences in ankle muscles and higher coherences in muscles involved in hip flexion and extension. Concerning rSTS effects, we expected to find that muscle fatigability would strengthen coherence more in healthy younger compared with older adults in synergistic and antagonistic muscles around the ankle and knee joints during gait.

## Results

### Participants characteristics and fatigability outcomes

Older and younger adults were not different in height, body mass, muscle mass, cognitive status, physical fitness, and trait level of perceived fatigability (Table 1).

**Table 1:** Participants' characteristics and scores on questionnaires.

	Younger	Older	p value
**Groups' characteristics**			
n (Male)	12 (7)	12 (7)	
Age (range), y	22 (20–25)	71 (66–77)	< 0.01
Height, cm	177.45 ± 2.65	173.13 ± 2.22	0.17
Body mass, kg	69.81 ± 3.29	73.92 ± 2.93	0.71
SPPB, scores	12 ± 0.00	12 ± 0.00	1.00
MFI, scores	38.18 ± 2.71	34.58 ± 2.83	0.52
**STS**			
Repetition	583.3 ± 50.12	134.12 ± 33.11	< 0.01
Duration, min	19.48 ± 1.71	4.47 ± 1.15	

Values are mean ± SE.

*SPPB* short physical performance battery, *MFI* multidimensional fatigue inventory, *STS* sit-to-stand.

Concerning muscle fatigability, older vs. younger performed ~ 4.3 × fewer STS repetitions (T_22_: 7.40, p < 0.01) (Table 1), accompanied by an increase in RPE from ~ 8 to ~ 18 in the two groups (F_1.22_: 346.24, p < 0.01, ${\upeta }_{p}^{2}$: 0.94, d: 1.90) (Table 2). There were Age and Time main effects for MVIF (F_1.22_: 10.38 and 32.07, p < 0.01 for all, ${\upeta }_{p}^{2}$: 0.32 and 0.59) (Table 2). The Age main effect indicated that older compared to younger adults were weaker (~ 37% lower MVIF, d: 0.82). After rSTS, MVIF decreased in the two age groups by ~ 16% (d: 0.37).

**Table 2:** Effects of age and rSTS on stride outcome.

	Time	Young adults	Older adults
		Mean ± SE	Mean ± SE
**Fatigability outcomes**			
MVIF, N	Before rSTS	676.25 ± 244.62	422.12 ± 133.13^a^
	After rSTS	563.78 ± 197.91^b^	354.83 ± 116.34^a,b^
Borg, score	Before rSTS	6.75 ± 1.22	8.42 ± 2.43
	After rSTS	19.25 ± 2.30^b^	18 ± 1.91^b^
**Stride outcomes**			
Stride length, cm	Before rSTS	132.67 ± 2.46	123.97 ± 2.23^a^
	After rSTS	130.25 ± 2.17	123.63 ± 2.61
Width, cm	Before rSTS	10.19 ± 1.30	10.3 ± 0.89
	After rSTS	10.17 ± 1.28	10.21 ± 0.76
Swing time, s	Before rSTS	0.39 ± 0.01	0.37 ± 0.01
	After rSTS	0.38 ± 0.01^b^	0.37 ± 0.01
Stance time, s	Before rSTS	0.72 ± 0.04	0.67 ± 0.04
	After rSTS	0.71 ± 0.04	0.67 ± 0.05
Cadence, steps/min	Before rSTS	108 ± 2.00	114 ± 2.00^a^
	After rSTS	109 ± 2.00	114 ± 2.00

Values are mean ± SE.

^a^Older ≠ Younger adults; ^b^After ≠ Before rSTS.

### Stride outcomes

Before rSTS, older vs. younger adults walked with 5% shorter strides (T_1.22=_2.11, p = 0.05, d: 0.86) and 5% higher cadence (T_1.22=_2.16, p = 0.04, d: 0.88) (Table 2).

After rSTS, swing time decreased by 3% (p < 0.05, d: 0.40) but rSTS did not affect any other stride metrics.

### Coherence and EMG-amplitude

*Supplementary Figures S1 and S2 detail the coherence data for all muscles and the two age groups before and after rSTS.

**Figure 1:**
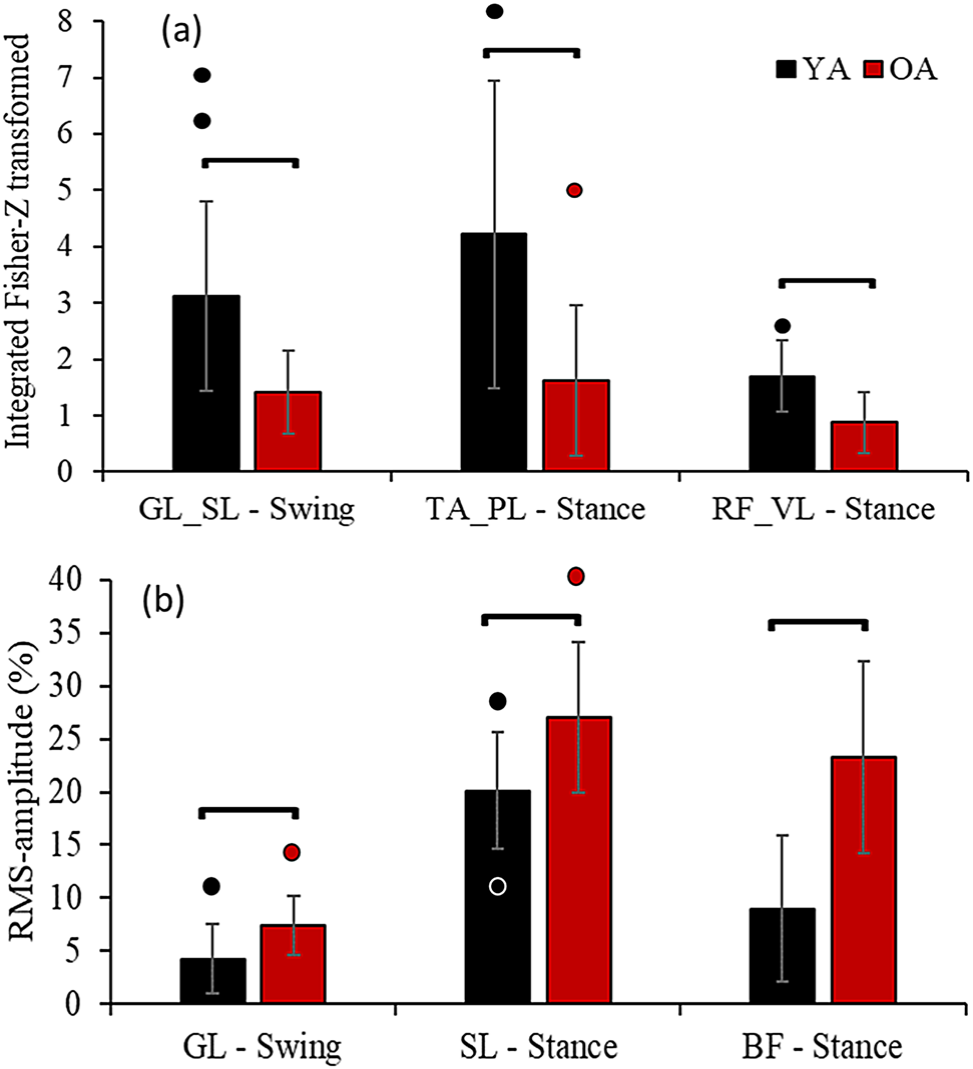
Mean ± standard deviation for the significant outcomes (dots are outliers). (**a**) Intermuscular coherence during walking between gastrocnemius lateralis (GL)-Soleus (SL) in swing phase and between tibialis anterior (TA)-peroneus longus (PL) and rectus femoris (RF)-vastus lateralis in stance phase. (**b**) RMS-amplitude for GL in swing, and SL and biceps femoris (BF) both in stance. *YA* younger adults, *OA* older adults.

Before rSTS, older vs. younger adults had ~ 55% lower GL-SL beta-band coherence (t_22_: 3.59, p < 0.01, d: 1.32) in late swing and 62% and 48% lower TA-PL (t_22_: 2.53, p = 0.02, d: 1.21) and RF-VL coherence (t_22_: 3.42, p < 0.01, d: 1.41) in early stance (Fig. 1a). Concerning RMS-amplitude, older vs. younger adults had ~ 75, 34 and 158% greater amplitude of GL in swing (t_22_: 3.11, p < 0.01, d: 1.05), and SL (t_22_: 2.65, p < 0.02, d: 1.09) and BF (t_22_: 4.44, p < 0.01, d: 1.77) (Fig. 1b).

**Figure 2:**
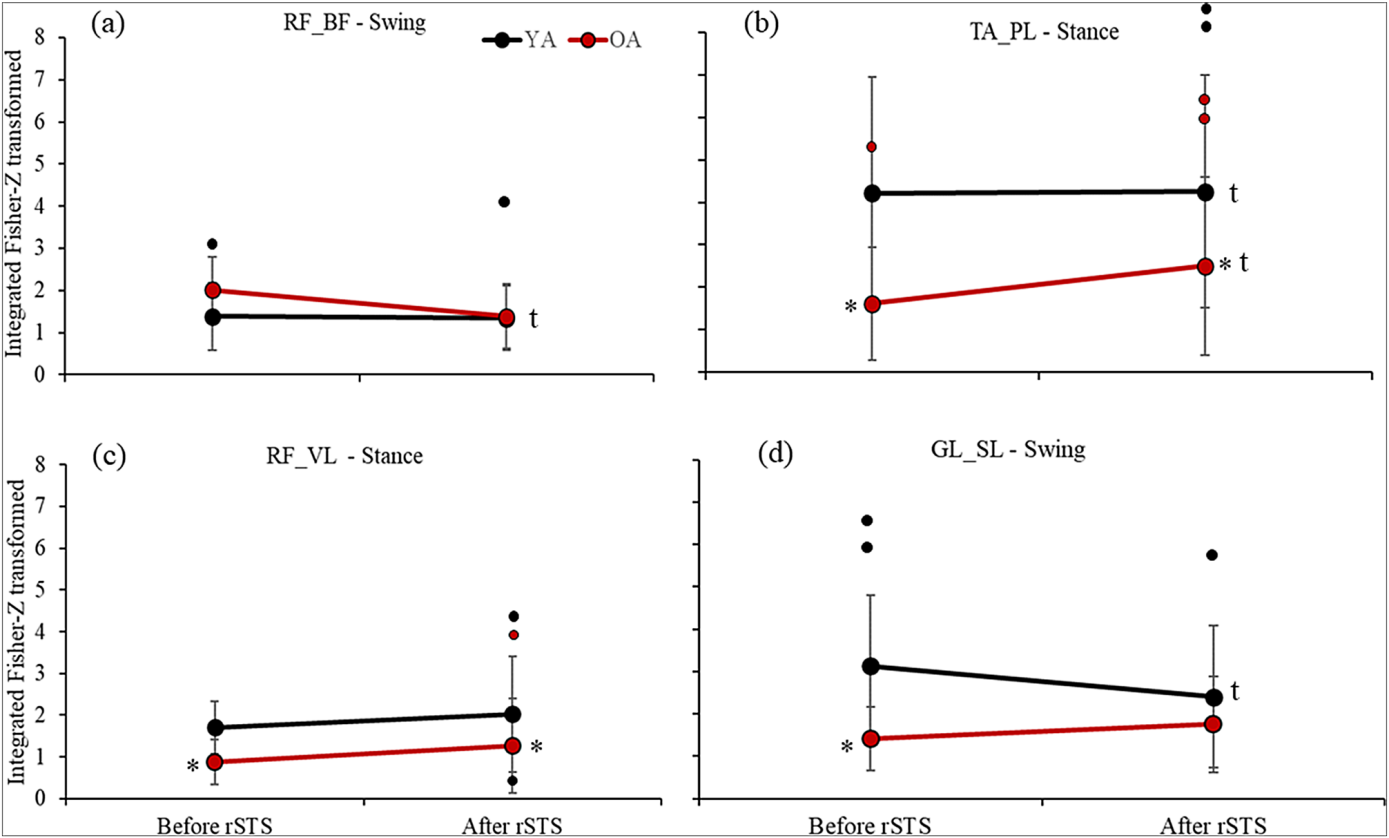
Mean ± standard deviation (dots are outliers) for the significant outcomes. (**a**) Intermuscular coherence between RF-BF in swing, (**b**) TA-PL and (**c**) RF-VL in stance, and (**d**) GL-SL in swing phase during treadmill walking. *Age-differences [Older (OA) ≠ Younger adults (YA)] indicated in post-hoc; ^t^time-differences [After ≠ Before repetitive sit-to-stand (rSTS)] indicated by Post-hoc.

After rSTS, RF-BF coherence decreased (F_1.22_: 6.68, p = 0.02, ${\upeta }_{p}^{2}$: 0.23) in late swing and TA-PL coherence increased in early stance phases (F_1.22_: 6.45, p = 0.02; ${\upeta }_{p}^{2}$: 0.23). RF-BF coherence decreased by 20% in swing phase (d: 0.35) and TA-PL coherence increased by 16% in stance phase (d: 0.21) (Figs. 2a,b) in the two age groups combined (time main effect). After rSTS, older vs. younger had ~ 43 less RF-VL (F_1.22_: 7.63, p = 0.01; ${\upeta }_{p}^{2}$: 0.26; d: 0.86) (Fig. 2c) and 44% less TA-PL coherences (F_1.22_: 4.75, p = 0.04; ${\upeta }_{p}^{2}$: 0.18; d: 0.76) in stance phase.

There was a Group by Time effect for GL-SL coherence in swing phase (F_1.22_: 5.03, p = 0.04; ${\upeta }_{p}^{2}$: 0.19) so that older vs. younger adults had 55% lower coherence at the baseline (p < 0.01; d: 1.32), without group-difference after rSTS (p > 0.05). This is because in younger adults, GL-SL coherence after rSTS decreased (23%) (p = 0.03, d: 0.43), while older adults GL-SL coherence after rSTS increased (23%, n.s.) (Fig. 2d).

**Figure 3:**
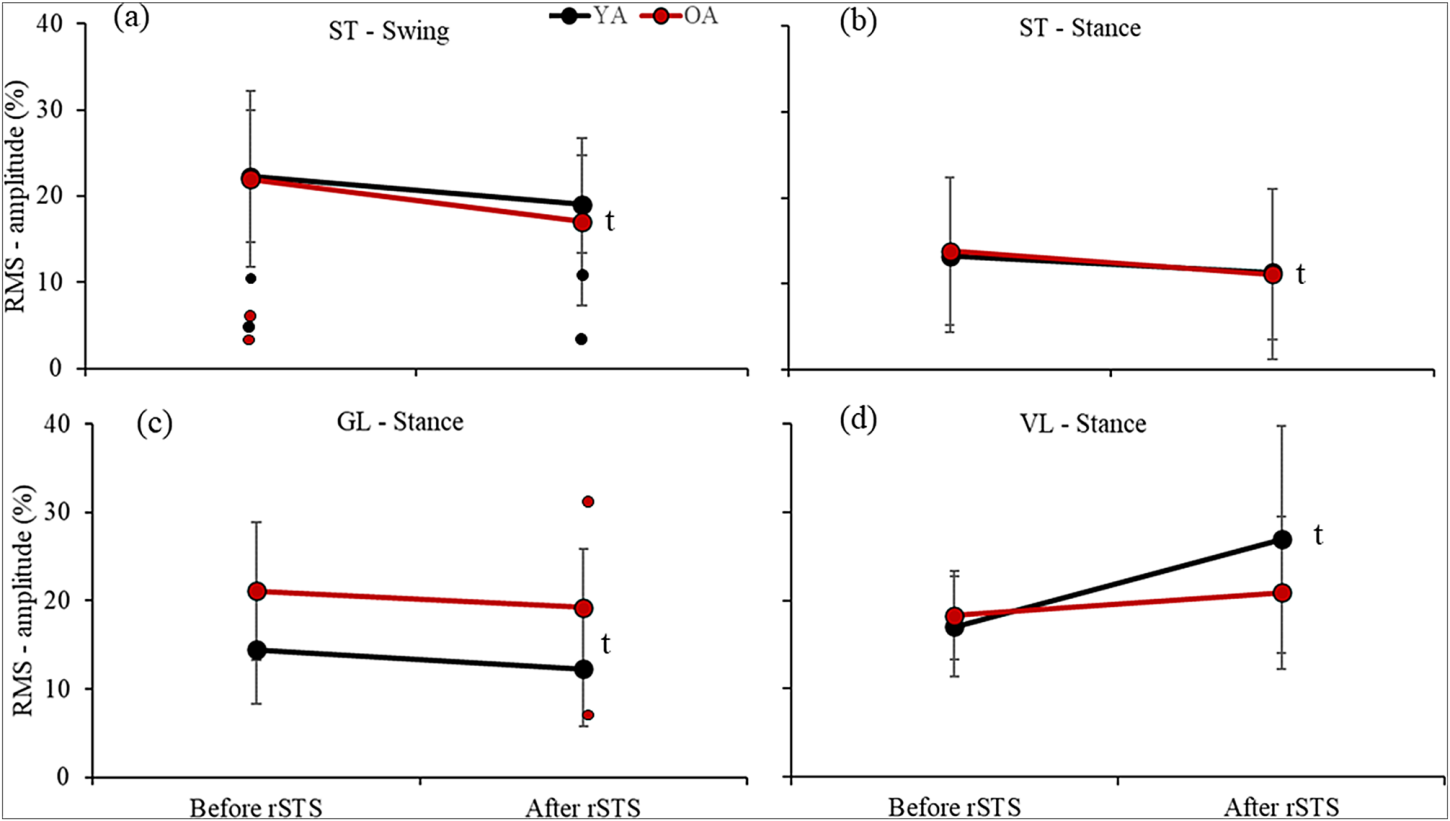
Mean ± standard deviation (dots are outliers) for the significant RMS-amplitude normalized by maximum amplitude on stride cycle. (**a**) ST in swing, (**b**) ST in stance, (**c**) GL in stance, and (**d**) VL in stance.; ^t^time-differences [After ≠ Before repetitive sit-to-stand (rSTS)] indicated by Post-hoc.

For RMS-amplitude, ANOVA revealed time main effect indicating that both age groups combined decreased (~ 19 and 17%) RMS of ST in both swing and stance phase (F_1.22_: 5.87 and 6.89, both p < 0.03; ${\upeta }_{p}^{2}$: 0.21 and 0.24; d: 0.42 and 0.26, respectively) (Fig. 3a,b), and GL in stance phase (F_1.22_: 4.49, p < 0.05; ${\upeta }_{p}^{2}$: 0.17; d: 0.30) (Fig. 3c), and increased (~ 35%) the RMS of VL in stance phase (F_1.22_: 8.22, p < 0.01; ${\upeta }_{p}^{2}$: 0.27; d: 0.73) (Fig. 3d).

**Figure 4:**
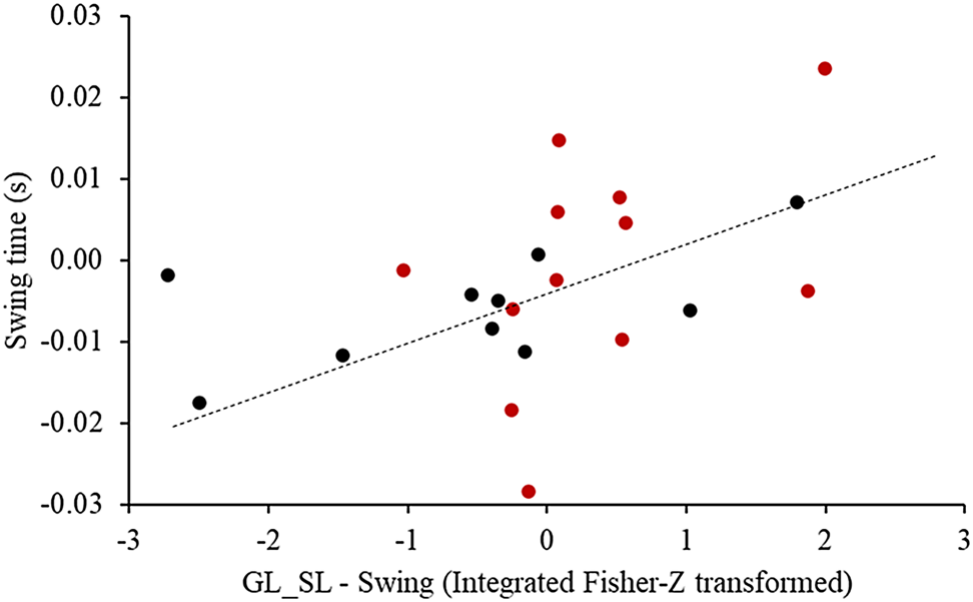
Significant correlations between absolute changes in GL-SL in swing phase and swing time due to muscle fatigability.

Before rSTS, there were no correlations between beta-band coherence in GL-SL in late swing, TA-PL and RF-VL early stance with MVIF, stride length, and cadence, and between RMS-amplitude of GL with GL-SL both in late swing (r = 0.20 to 0.38; p > 0.05) (Supplementary Figure S3). rSTS-induced decreases in GL-SL coherence during Swing phase correlated with decreases in swing time (r = 0.59, p < 0.01) (Fig. 4). Absolute changes in variables did not correlate with absolute changes in coherence after rSTS (r = 0.02 to 0.19, p > 0.05) (Supplementary Figure S4).

## Discussion

We examined the effects of age on beta-band coherence during treadmill walking before and after experimentally induced muscle fatigability. The data confirmed the hypothesis of age-specific reductions in beta-band coherence between specific ankle muscles during gait. Against the hypothesis, we did not find the expected age-related increases in coherence among hip muscles. Also against the hypothesis, experimentally induced muscle fatigability did not strengthen the beta-band coherence across synergistic and antagonistic muscle pairs around the ankle and knee joints during gait in an age-specific manner. We, however, observed that older adults developed a compensatory neural strategy by increasing the TA-PL and GL-SL beta-band coherence in response to experimentally induced fatigability. Together, age affects the oscillatory coupling between synergistic muscle pairs, delivered presumably via corticospinal tracts, during treadmill walking. Muscle fatigability elicits age-specific changes in the common fluctuations in muscle activity, which could be interpreted as a compensation for muscle fatigability to maintain gait performance.

Concerning the age-effects on beta-band coherence during treadmill walking, we found that beta-band coherence was 44–62% lower in TA-PL, GL-SL, and RF-VL in older vs. younger adults during gait (Fig. 1). The age-effects on intermuscular coherence agree with previous intramuscular coherence data indicating an age-typical reduction in coherence between proximal and distal TA^1,9^. However, our data extend those previous findings^1,9^ by showing age-related reductions in coherence between synergistic ankle and knee muscle pairs. The age-related reductions in coherence GL-SL, TA-PL, and RF-VL may be related to the age-typical decline/weakness of common presynaptic inputs to the motor neuron pool^17^ that potentially reflected in a reduced oscillatory behavior between muscles activated during walking^1,10,39,40^. Indeed, brain data suggest enhanced inhibitory cortical control in old age that is associated with reduced oscillatory activation of motoneurons^41^. The inhibitory control may reflect the number and strength of neural commands from the cortex to the muscles, as confirmed by the reduced cortico-muscular coherence^1,39^. Because it is widely believed that intermuscular beta-band coherence may arise from shared inputs via branched corticospinal pathways^16,17,39,42^, reduced beta-band coherence in older adults might be indicative of inefficiency in the CNS to reduce the dimensionality by sending common drives to muscles that exert similar functions^17^. The reductions in beta-band coherence, therefore, may reflect a decline in motor performance^43^. Indeed, the reduced beta-band coherence in the ankle and knee synergistic extensor muscles in older adults were accompanied by altered stride pattern, i.e., ~ 10 cm shorter stride length and 6 steps/min higher cadence (Table 2), although the correlation failed in showing any association.

Curiously, our results indicated age-differences only for synergistic (BF-VL, TA-PL, and GL-SL, Fig. 2) but not for antagonistic muscle pairs coherence (RF-BF and TA-PL). Such findings only in synergistic and not in antagonistic muscle pairs are reasonable^44^, considering synergistic/antagonistic muscle cooperation during the gait cycle. For example, the gastrocnemius and soleus act together to stabilize the ankle and to absorb the energy generated by the braking impulse at heel strike^45^. Reduced beta-band coherence in these muscles can imply in event-related desynchronization during gait, implying less muscle coordination to stabilize the ankle joint during the braking impulse^46^. The data also suggest a reduced oscillatory synchronization for TA and PL (62%) during early stance phase (Fig. 2) and for RF and VL (48 and 43%) in both late swing and early stance phases in older compared with younger adults (Figs. 1, 2b,c). These two muscle pairs work to decelerate the center of mass and stabilize the joints, keep the toe-clearance, and maintain the natural stance movement mechanics^45^. A lack of coherence for the antagonistic RF-BF and TA-GL muscle pairs might suggest that independent neural commands are being delivered to each muscle. This idea is further supported by previous data showing lower intermuscular coherence in antagonistic vs. synergistic muscle pairs^44,47^, and by studies that indicate that such antagonistic muscles activate in different moments of the gait cycle^45^. Although we observed age-related changes in beta-band coherence in synergistic muscles, there was an absence of accentuated antagonistic intermuscular beta-band coherence in healthy older adults, such as those observed in spinal cord injury population^16^. Possibly, it might be due to a relatively preserved pyramidal tract in healthy old vs. spinal cord injury population.

Our findings extend the current literature^1,9^ to infer an age-related decline in the oscillatory coupling between muscles spanning lower extremity joints (ankle, knee and hip). The age-related decrease in common neural input occurred in synergistic lower extremity muscles. Curiously, such decreases seem not to be related to the age-typical reductions in mechanical output at the ankle and the increases in mechanical work generation at the hip joint^6^. Reduced RF-VL, TA-PL, and GL-SL beta-band coherence in older vs. younger adults may be related to general age-related reductions in the common neural drive via corticospinal tracks to synergistic muscle pairs during treadmill walking. Such age-specific reductions in beta-band coherence between synergistic muscle pairs before rSTS were further modulated in order to compensate for the muscle fatigability in older adults, thus keeping the stride metrics of gait performance unchanged (Table 2).

With regards to the fatigability effects on beta-band coherence, we expected that muscle fatigability would strengthen coherence around the ankle and knee joints during gait more so in healthy younger than in older adults. We, however, observed an age-specific modulation intermuscular coherence in ankle muscle pairs after muscle fatigability. Such a modulation suggests that older adults developed a compensatory strategy in response to the decline in motor performance induced by rSTS to maintain neural control of lower extremity muscles during gait, resulting in unchanged stride metrics of gait performance.

Muscle fatigability tends to increase intermuscular beta-band coherence^25,26,28^. This increase may be interpreted as a compensatory mechanism of CNS to increase the strength of the common neural drive to muscles to maintain a target force or performance^26,28^. Therefore, our result of subtle increases in TA-PL beta-band coherence in stance phase is in line with the expected changes in coherence after rSTS. In contrast and unexpectedly, both age groups revealed a slight decrease in RF-BF intermuscular coherence in the stance phase. Additionally, the particular age adaptation in synergistic GL-SL intermuscular coherence in stance phase was also unforeseen.

Perhaps, the most intriguing result was the age-specific adaptation in intermuscular coherence in ankle muscle pairs after muscle fatigability. While there was 23% decrease in GL-SL common drive in younger adults, older adults showed 23% increase in this coherence after muscle fatigability (Fig. 2d). In addition, the main effect indicated a 16% increase in TA-PL coherence in the two age groups after rSTS, driven by the 55 vs. 1% increase in older and younger adults (Fig. 2b). Thus, there was a trend for age by time interaction in this important outcome (p = 0.075; ${\upeta }_{p}^{2}$: 0.14). One possible interpretation for age-differences in these intermuscular coherences is compensation for the decline in motor performance induced by rSTS. Age-specific increase in these coherences may reflect higher susceptibility to fatigability^19^ and/or an age-related increase in demand of walking after muscle fatigability^5^. Indeed, while both age groups showed similar decreases in MVIF, older compared with younger adults performed remarkably fewer STS repetitions (Table 2).

Maximal voluntary contraction-induced fatiguability was associated with physiological tremor, which was interpreted as an increase in afferent feedback from muscle spindles to the motor units^30^. The increase in feedback presumably strengthens the correlated inputs to motor units as fatigability progresses^30^. Our results partially support this finding because while we observed increases in beta-band coherence, the increase in alpha-band coherence was minimal as were the correlations between these two coherences in older adults (data not shown). Concerning the higher walking demand, previous data showed that the metabolic cost of gait was ~ 20% higher, which was related to 67% higher agonist muscle activation and 153% greater antagonist coactivation during walking^5^. Together, the higher muscle fatigability effects and higher walking demand in older compared with younger adults seem to have elicited compensatory neural adaptations to muscle fatigability, making it possible for older adults to continue to walk.

The small decreases in intermuscular coherence between antagonist muscle pairs (RF-BF) were somewhat unexpected. Normally such coherences tend to increase rather than decrease after muscle fatigability. The increases are attributed to enhanced cortical excitability to compensate for muscle fatigability^25,31^, normally associated with higher coactivation. Although high cortical excitability due to muscle fatigability is a physiological explanation for both increases in muscle amplitude and coherence^25,31^, we recognize that increases in intermuscular coherence do not necessarily mean an increase in coactivation. In addition, both increased coactivation and antagonistic intermuscular coherence could impair muscle coordination and motor performance^4,26^.

The changes in beta-band coherence after experimentally induced fatigability were not accompanied by any meaningful changes in stride outcomes during treadmill walking (Table 2). Our results indicating only small changes in swing time (0.01 s) for young adults reiterate the minimal effects of age and fatigability on strides outcomes observed previously^35^. Possibly, those changes in beta-band coherence may represent a compensatory neural strategy in response to experimentally induced fatigability to maintain the strides metrics unchanged.

As limitations, albeit intermuscular coherence during gait has recently received attention, the interpretation of such data is not straightforward. It is because relating individual variation in intermuscular coherence to variation in gait behavior due to age or perturbations, as done here, has been moderately successful at best^1,9^. In addition, although our findings revealed large ANOVA effects sizes (${\upeta }_{p}^{2}$) and significant effects in selected coherence outcomes, the statistical powers were lower (between 60 and 79%) than the ideal levels of power (80%) for such outcomes (details in Supplementary analysis), suggesting that the study might have been underpowered^48^. Furthermore, the influence of EMG rectification on coherence has been discussed^46,49,50^. We opted to use EMG rectification procedure because it amplifies temporal information about motor unit action potentials during submaximal tasks, as gait^46,49–51^, and helps with the interpretation of the results with the literature^1,10,39,42,46,47,52^. The Fourier transformation (a standard protocol used in coherence analyses^1,10,42,46^) is valid for stationary signals, a condition unlikely to be met in gait. To address this limitation, we analyzed coherence in short time windows during swing and stance phases of gait. Similarity of temporal features of gait before and after fatigability in younger and older adults further decreased the likelihood of non-stationarity in the signal (Table 2). In addition, we recognize the limitation of coherence analyses because the cortical or spinal origin of coherence is not unambiguous and could be affected by amplitude cancellation and cross-talk^53^. However, we have made every effort to minimize such effects by computing RMS-amplitude and verifying the absence of correlations between absolute values and changes between RMS-amplitudes and coherence for the significant outcomes. While treadmill vs. overground walking has the advantage of examining gait under a standardized condition, treadmill walking requires reactive rather than feed-forward adjustments of walking. Treadmill makes walking unvarying^54,55^ and, compared with overground walking, it reduces muscle activation^56^ and coherence^46^. Therefore, the age and fatigability effects on intermuscular coherence might be more overt during overground walking.

The current study contributes to the understanding of how health aging brings about adaptations muscle activation during gait and how such adaptations might turn into compensations to help maintain gait performance on the treadmill. In conclusion, age affects the oscillatory coupling between synergistic muscle pairs, delivered presumably via corticospinal tracts, during treadmill walking. Muscle fatigability elicits age-specific changes in the common fluctuations in muscle activity, which could be interpreted as a compensation for muscle fatigability to maintain gait performance.

## Methods

### Participants

Healthy younger (n = 12, age range 20–25 years, 5F) and older (n = 12, age range 66–77 years, 5F) participants completed the study. Inclusion criteria were: age < 25 or > 65 years and either gender. Exclusion criteria were: lower limb musculoskeletal injury or surgery that could affect walking ability; inability to walk unassisted on a treadmill; self-reported pain in the lower extremities, and neurological or cardiac diseases. The procedures of this study were performed in accordance with the Declaration of Helsinki^57^ and were approved by the Ethical Committee of the Department of Human Movement Sciences, University Medical Center Groningen (#ECB2017.06.12_1). All participants signed the informed consent document before testing.

### Procedures

**Figure 5:**
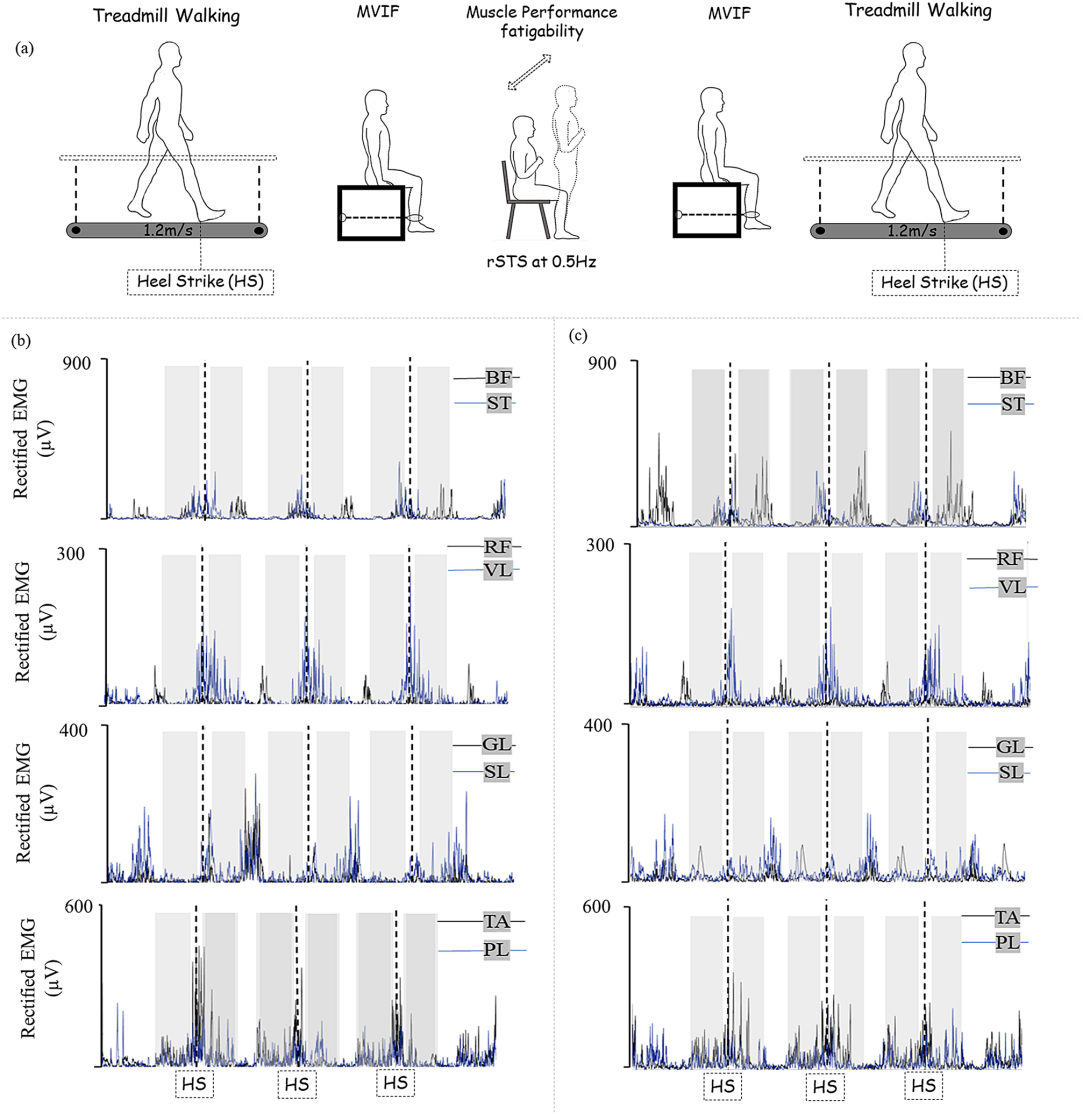
(**a**) Experimental setup and EMG data (data high-pass filtered 5 Hz using a second-order Butterworth filter, full-wave rectified using Hilbert transform data) of one younger subject before (**b**) and after (**c**) rSTS, considering 3 out of 100 heel strike (HS) events (dashed lines). Each plot on panel (**b**,**c**) consists of two muscles (represented in black and blue lines). Shaded areas illustrate the 350 ms of analyses for late swing (− 400 to − 50 ms before heel strikes) and early stance (50–400 ms after heel strikes) phases. *MVIF* maximum voluntary isometric force, *rSTS* repetitive sit-to-stand task, *HS* heel strike, *BF* biceps femoris, *ST* semitendinosus, *RF* rectus femoris (RF), *VL* vastus lateralis, *GL* gastrocnemius lateralis, *SL* soleus, *TA* tibialis anterior, *PL* peroneus longus (PL).

The cognitive status, physical fitness, and trait level of perceived fatigability were assessed by the Mini-Mental State Examination^58^, Short Physical Performance Battery^59^, and Multidimensional Fatigue Inventory^60^, respectively. Participants then walked on a treadmill for 3 min, performed a knee extension to measure the maximal voluntary isometric force (MVIF), executed the rSTS to induce muscle performance fatigability, performed an MVIF, and walked on the treadmill (Fig. 5), described in detail previously^35^.

### Data acquisition

Participants, wearing a harness, walked on a treadmill instrumented with two force plates (Motek, Amsterdam, NL) for 3 min before and after rSTS. They were asked not to hold onto the handrails and to look at a target (letter X) displayed on the wall in front of them 5 m away. We assessed gait in an instrumented treadmills to be able to record consecutive steady-state stride cycles, exactly controlling walking speed^61^. Walking speed was fixed at 1.2 m/s. A fixed walking speed was chosen to avoid a potential effect of speed on intermuscular coherence, and 1.2 m/s was selected to be similar to the comfortable speed for both young and older groups^62,63^. We collected 3-D ground reaction forces and moments of force under each leg at 1 kHz using a D-Flow acquisition software (Motek, Amsterdam, NL, USA).

To record EMG activity during walking, 8 wireless sensors (dimensions: 37 × 26 × 15 mm, electrode material: silver; Trigno Wireless System, Delsys, Natick, MA, USA) were placed unilaterally on the following muscles: soleus (SL), gastrocnemius lateralis (GL), TA, peroneus longus (PL), vastus lateralis (VL), rectus femoris (RF), biceps femoris (BF), and semitendinosus (ST) according to Surface Electromyography for the Non-Invasive Assessment of Muscles (SENIAM) conventions^64^. EMG signals were sampled at 2 kHz. The areas where the sensors were placed, body hair was removed, and the skin was cleaned with alcohol. Treadmill and EMG data acquisition were electronically synchronized with a custom-built timer and event generator.

MVIF was assessed on a custom-built dynamometer. Participants, seated in a chair with the knee and hip in 90° of flexion (measured by a Goniometer), had the non-dominant lower leg strapped to the chairs’ lever arm (~ 10 cm up to lateral malleolus). We instructed participants to contract the quadriceps as rapidly and forcefully as possible and to maintain force generation for 5 s. MVIF was determined as the peak of force.

To induce muscle fatigability, participants performed the rSTS at 0.5 Hz in a standard dimension chair (0.43 × 0.41 × 0.42 m). The protocol was stopped either when participants were unable to continue or after 30 min. Task duration and the number of repetitions were recorded. Before and after rSTS, we asked participants to rate their perceived exertion (RPE) on a 6–20 Borg Scale^65^.

### Data analysis

Data were analyzed using custom MATLAB routines (version r2018; MathWorks, Natick, MA, USA). Ground reaction forces and moments of force were filtered with a 15-Hz low-pass second-order zero-phase Butterworth filter. Heel contact and toe-off were determined using a threshold of 50 N vertical ground reaction force. Using heel strike and toe-off, we calculated stride length, step width, stance time, swing time, and cadence^66^ using the average of the middle 100 strides performed with the dominant leg during 3 min of treadmill walking before and after rSTS.

EMG data were visually inspected for minimizing noise and artifacts. Then, the data were high-pass filtered at 5 Hz using a second-order Butterworth filter, full-wave rectified using Hilbert transform. Then, EMG data were downsampled to 1 kHz to obtain the same frequency of the force data plate.

### Coherence calculation

We calculated the frequency-domain coupling between two EMG signals acquired during the treadmill walking before and after rSTS. For EMG coherence, we set two windows of 350 ms for analysis^1^: late swing phase (from − 400 to − 50 ms before the heel strike) and early stance phase (from 50 to 350 ms after the heel strike) (Fig. 5b,c). In both phases, we calculated coherence considering 4 synergistic muscle pairs: BF-ST, RF-VL, GL-SL, TA-PL, and 2 antagonistic muscle pairs: RF-BF; TA-GL.

To calculate intermuscular coherence, we first determine the auto-spectra of each muscle (*f*_*xx*_ and *f*_*yy*_) and cross-spectrum (*f*_*xy*_) of the muscle pairs (TA-PL, GL-SL, RF-VL, BF-ST, TA-GL, and RF-BF) using Welch’s periodogram method^25,67^. For each of the 100 strides selected, estimates were obtained using 350 ms window (separately for late swing and early stance phases), nonoverlapping data segments, resulting in a frequency of 2.86 Hz of resolution. Spectral estimates of individual strides were then averaged across the 100 strides (same used to calculate the stride outcomes) and used to calculate coherence. Intermuscular coherence was calculated by the squared modulus of cross-spectrum divided by the product of the two auto-spectrum for each frequency (λ)^11^:

$$c\left(\lambda \right)=\frac{{\left|{f}_{xy}\left(\lambda \right)\right|}^{2}}{{f}_{xx}\left(\lambda \right)\cdot {f}_{yy}\left(\lambda \right)}$$

where *f*_*xx*_ and *f*_*yy*_ are the auto-spectra of each muscle, and *f*_*xy*_ is the cross-spectrum of the muscle pairs (TA-PL, GL-SL, RF-VL, BF-ST, TA-GL, and RF-BF). Values of coherence range from 0 (absent) to 1 (completely correlated)^11^ and in a frequency range of 0–55 Hz. For each subject, intermuscular coherence was significant when it exceeded the confidence limit for the number of segments (L) used to estimate the spectrum^11,68^, as given:

$$1-{\left(\alpha \right)}^{\frac{1}{L-1}}$$

where *α* = 0.05 and L is the number of strides (N = 100) used in the analysis.

For each subject and muscle pair, we ascertained from the cumulant density plots that high coherences were accompanied by near zero-lag synchronization suggesting that cross-talk did not affect coherences^16,69^. To compare coherence between age groups and time (before and after rSTS), intermuscular coherence of each subject was combined into pooled estimates for age groups for each walking phase before and after rSTS. Coherence estimates were Fisher transformed before pooling to stabilize variance^68,69^. The amount of coherence was calculated by the cumulative sum (area) on the range of the beta-band frequency domain (15–35 Hz) for each age group, phase, and before and after rSTS.

### RMS-amplitude

For EMG amplitude, the raw EMG data were band-pass filtered (20–450 Hz) using a fourth-order Butterworth filter, full-wave rectified using Hilbert transform, and downsampled to 1 kHz. For each stride, the RMS-amplitude for each muscle was calculated considering the 350 ms windows at late swing and early stance phase, and the values were expressed as a percentage of the peak amplitude within the stride cycle considering the walking condition before rSTS. RMS-amplitudes were averaged across the 100 strides.

### Statistical analysis

Statistical analyses were performed in SPSS for Windows (Version 25, IBM, Armonk, NY, USA). When Shapiro–Wilk tests revealed non-normal distribution, data were log-transformed for further comparisons using t-test or ANOVA. T-tests were used to compare the effects of age on Groups’ characteristics (age, height, body mass, SPPB, MFI), rSTS duration, and the number of STS repetitions, stride outcomes, RMS-amplitude, and beta-band coherence before rSTS. To compare the effects of experimentally induced fatigability, we conducted a repeated-measures ANOVA with as between factor Age (younger vs. older adults) and within factor Time (time 1: before rSTS vs. time 2: after rSTS) for MVIF, strides outcomes, RMS-amplitude, and beta-band coherence. ANOVA effect size was estimated using partial eta squared (${\upeta }_{p}^{2}$) with ${\upeta }_{p}^{2}$ <0.01, 0.06 and > 0.13 (that reflect in ƒ = 0.1, 0.25 and > 0.40) as small, medium and large effects size^70^. If interactions were significant, post-hoc comparisons for each factor were made, and the level of significance was adjusted for multiple comparisons by using Bonferroni correction. For post-hoc and t test comparisons, Cohen’s d was calculated, and we interpreted 0.21–0.50, 0.51 to 0.79 and > 0.79 as small, medium and large effect sizes (d), respectively^70^. In G-power^71^, post-hoc power analyses were calculated, with an assumed Type I error of 0.05, considering the current sample size and the ${\upeta }_{p}^{2}$ for each outcome. The power calculation resulted in a range of Type II error rate of 0.21–0.45 (55–79% statistical power).

As additional analyses, we computed correlations between the outcomes (beta-band coherence, MVIF, stride metrics). Before rSTS, a Spearman’s correlation was computed between absolute values of the outcomes that indicated significant age differences (p < 0.05). We also computed Spearman’s correlations between absolute changes (delta, after–before rSTS) in the outcomes that revealed a significant Time effect. Additionally, if a t test or ANOVA revealed significant differences in RMS-amplitude between two muscles included in coherence analyses, we computed Spearman’s correlations between absolute values (before rSTS) and changes (delta) RMS-amplitude and coherence. Such correlations would tell us if RMS-amplitude was driving coherence.

## Supplementary information

Supplementary Information 1

Supplementary Information 2

Supplementary Information 3

## Data Availability

All data and additional figures generated in this study are included as Supplementary Information files.
