# Supplementary material for: Age-specific modulation of intermuscular beta coherence during gait before and after experimentally induced fatigue
Source: Sci Rep. 2020 Sep 28;10:15854. doi: 10.1038/s41598-020-72839-1 (PMC7522269; doi:10.1038/s41598-020-72839-1)
Supplement: Supplementary file 1 — Supplementary Information 1 [file 41598_2020_72839_MOESM1_ESM.docx]

**Supplementary information – Supplementary analysis**

**Age-specific modulation of intermuscular beta coherence during gait before and after** **experimentally induced fatigue**

Paulo Cezar Rocha dos Santos, ­­­­Claudine J. C. Lamoth, Fabio Augusto Barbieri, Inge Zijdewind, Lilian Teresa Bucken Gobbi, Tibor Hortobágyi

Power analyses were conducted in G-power (v. 3.1.9.2) considering ANOVA outcomes (figure 1). For each outcome, post-hoc power analysis was computed considering the total sample sizes, number of groups, measurements, and ƒ effect size (based on partial eta-square ($\eta_{p}^{2}$) from the SPSS output). Details are bellow.


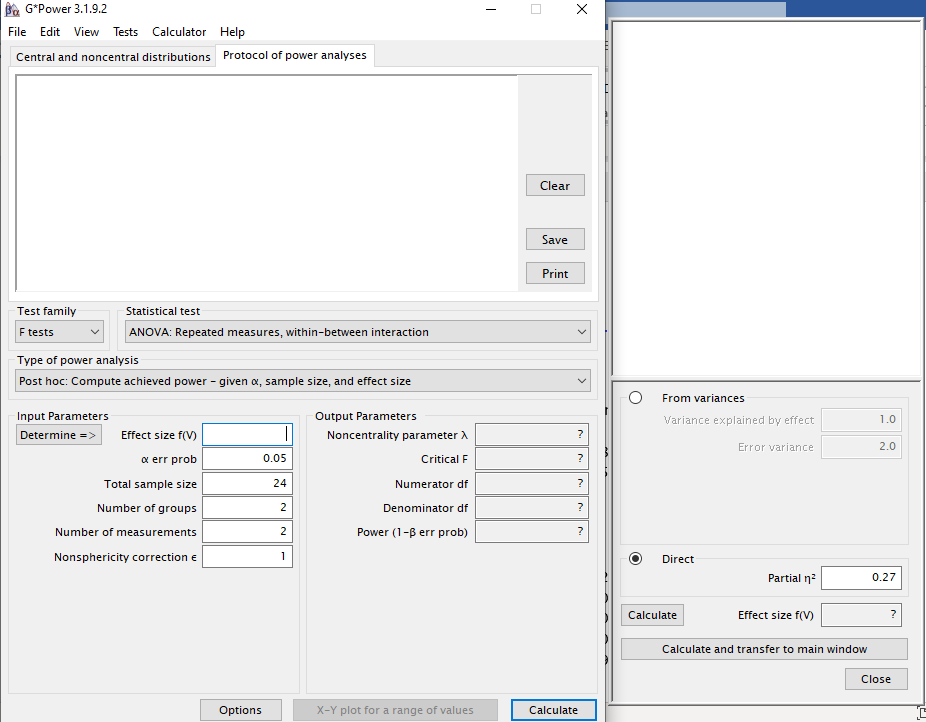


**Figure 1.** G*power overview.

[1] *– MVIF (AGE)*

**F tests -** ANOVA: Repeated measures, within-between interaction

**Analysis:** Post hoc: Compute achieved power

**Input:** Effect size f(V) ($\eta_{p}^{2}$: 0.32) = 0.6859943

α err prob = 0.05

Total sample size = 24

Number of groups = 2

Number of measurements = 2

Nonsphericity correction ε = 1

**Output:** Noncentrality parameter λ = 11.2941163

Critical F = 4.3009495

Numerator df = 1.0000000

Denominator df = 22.0000000

Power (1-β err prob) = 0.8944954

[2] *– MVIF (TIME)*

**F tests -** ANOVA: Repeated measures, within-between interaction

**Analysis:** Post hoc: Compute achieved power

**Input:** Effect size f(V) ($\eta_{p}^{2}$: 0.59) = 1.1995934

α err prob = 0.05

Total sample size = 24

Number of groups = 2

Number of measurements = 2

Nonsphericity correction ε = 1

**Output:** Noncentrality parameter λ = 34.5365838

Critical F = 4.3009495

Numerator df = 1.0000000

Denominator df = 22.0000000

Power (1-β err prob) = 0.9998666

[3] *– RPE (TIME)*

**F tests -** ANOVA: Repeated measures, within-between interaction

**Analysis:** Post hoc: Compute achieved power

**Input:** Effect size f(V) ($\eta_{p}^{2}$: 0.94) = 3.9581140

α err prob = 0.05

Total sample size = 24

Number of groups = 2

Number of measurements = 2

Nonsphericity correction ε = 1

**Output:** Noncentrality parameter λ = 376

Critical F = 4.3009495

Numerator df = 1.0000000

Denominator df = 22.0000000

Power (1-β err prob) = 1.0000000

[4] *– RF_BF_SWING (TIME)*

**F tests -** ANOVA: Repeated measures, within-between interaction

**Analysis:** Post hoc: Compute achieved power

**Input:** Effect size f(V) ($\eta_{p}^{2}$: 0.23) = 0.5465357

α err prob = 0.05

Total sample size = 24

Number of groups = 2

Number of measurements = 2

Nonsphericity correction ε = 1

**Output:** Noncentrality parameter λ = 7.1688305

Critical F = 4.3009495

Numerator df = 1.0000000

Denominator df = 22.0000000

Power (1-β err prob) = 0.7254815

[5] *– TA_PL_STANCE (TIME)*

**F tests -** ANOVA: Repeated measures, within-between interaction

**Analysis:** Post hoc: Compute achieved power

**Input:** Effect size f(V) ($\eta_{p}^{2}$: 0.23) = 0.5465357

α err prob = 0.05

Total sample size = 24

Number of groups = 2

Number of measurements = 2

Nonsphericity correction ε = 1

**Output:** Noncentrality parameter λ = 7.1688305

Critical F = 4.3009495

Numerator df = 1.0000000

Denominator df = 22.0000000

Power (1-β err prob) = 0.7254815

[6] *– TA_PL_STANCE (AGE)*

**F tests -** ANOVA: Repeated measures, within-between interaction

**Analysis:** Post hoc: Compute achieved power

**Input:** Effect size f(V) ($\eta_{p}^{2}$: 0.18) = 0.4685213

α err prob = 0.05

Total sample size = 24

Number of groups = 2

Number of measurements = 2

Nonsphericity correction ε = 1

**Output:** Noncentrality parameter λ = 5.2682930

Critical F = 4.3009495

Numerator df = 1.0000000

Denominator df = 22.0000000

Power (1-β err prob) = 0.5926918

[7] *– GL_SL_Swing (AGE*TIME)*

**F tests -** ANOVA: Repeated measures, within-between interaction

**Analysis:** Post hoc: Compute achieved power

**Input:** Effect size f(V) ($\eta_{p}^{2}$: 0.186) = 0.4780180

α err prob = 0.05

Total sample size = 24

Number of groups = 2

Number of measurements = 2

Nonsphericity correction ε = 1

**Output:** Noncentrality parameter λ = 5.4840290

Critical F = 4.3009495

Numerator df = 1.0000000

Denominator df = 22.0000000

Power (1-β err prob) = 0.6098315

[8] *– RF_VL_STANCE (AGE)*

**F tests -** ANOVA: Repeated measures, within-between interaction

**Analysis:** Post hoc: Compute achieved power

**Input:** Effect size f(V) ($\eta_{p}^{2}$: 0.26) = 0.5927490

α err prob = 0.05

Total sample size = 24

Number of groups = 2

Number of measurements = 2

Nonsphericity correction ε = 1

**Output:** Noncentrality parameter λ = 8.4324330

Critical F = 4.3009495

Numerator df = 1.0000000

Denominator df = 22.0000000

Power (1-β err prob) = 0.7925173

[9] *– RMS_ ST_SWING (TIME)*

**F tests -** ANOVA: Repeated measures, within-between interaction

**Analysis:** Post hoc: Compute achieved power

**Input:** Effect size f(V) ($\eta_{p}^{2}$: 0.21) = 0.5155800

α err prob = 0.05

Total sample size = 24

Number of groups = 2

Number of measurements = 2

Nonsphericity correction ε = 1

**Output:** Noncentrality parameter λ = 6.3797457

Critical F = 4.3009495

Numerator df = 1.0000000

Denominator df = 22.0000000

Power (1-β err prob) = 0.6752646

[10] *– RMS_ST_STANCE (TIME)*

**F tests -** ANOVA: Repeated measures, within-between interaction

**Analysis:** Post hoc: Compute achieved power

**Input:** Effect size f(V) ($\eta_{p}^{2}$: 0.24) = 0.5619515

α err prob = 0.05

Total sample size = 24

Number of groups = 2

Number of measurements = 2

Nonsphericity correction ε = 1

**Output:** Noncentrality parameter λ = 7.5789477

Critical F = 4.3009495

Numerator df = 1.0000000

Denominator df = 22.0000000

Power (1-β err prob) = 0.7489718

[11] *– RMS_GL_STANCE (TIME)*

**F tests -** ANOVA: Repeated measures, within-between interaction

**Analysis:** Post hoc: Compute achieved power

**Input:** Effect size f(V) ($\eta_{p}^{2}$: 0.17) = 0.4525696

α err prob = 0.05

Total sample size = 24

Number of groups = 2

Number of measurements = 2

Nonsphericity correction ε = 1

**Output:** Noncentrality parameter λ = 4.9156618

Critical F = 4.3009495

Numerator df = 1.0000000

Denominator df = 22.0000000

Power (1-β err prob) = 0.5635139

[12] *– RMS_VL_STANCE (TIME)*

**F tests -** ANOVA: Repeated measures, within-between interaction

**Analysis:** Post hoc: Compute achieved power

**Input:** Effect size f(V) ($\eta_{p}^{2}$: 0.27) = 0.6081636

α err prob = 0.05

Total sample size = 24

Number of groups = 2

Number of measurements = 2

Nonsphericity correction ε = 1

**Output:** Noncentrality parameter λ = 8.8767111

Critical F = 4.3009495

Numerator df = 1.0000000

Denominator df = 22.0000000

Power (1-β err prob) = 0.8125186
