# Supplementary material for: Age-specific modulation of intermuscular beta coherence during gait before and after experimentally induced fatigue
Source: Sci Rep. 2020 Sep 28;10:15854. doi: 10.1038/s41598-020-72839-1 (PMC7522269; doi:10.1038/s41598-020-72839-1)
Supplement: Supplementary file 3 — Supplementary Information 3 [file 41598_2020_72839_MOESM3_ESM.docx]

**Supplementary information – Supplementary figures 1 – 4**

**Age-specific modulation of intermuscular beta coherence during gait before and after** **experimentally induced fatigue**

Paulo Cezar Rocha dos Santos, ­­­­Claudine J. C. Lamoth, Fabio Augusto Barbieri, Inge Zijdewind, Lilian Teresa Bucken Gobbi, Tibor Hortobágyi

**Supplementary Fig 1.** Means (lines) and standard errors (shaded areas) of intermuscular coherence for all muscle pairs during 100 swing phase events. Younger adults (YA) = black; Older adults (OA) = red; Before = continuous lines; after rSTS = dashed lines. Shaded rectangle = beta-band frequency (15 to 35hz).

**Supplementary Fig 2.** Means (lines) and standard errors (shaded areas) of intermuscular coherence for all muscle pairs during 100 stance phase events. Younger adults (YA) = black; Older adults (OA) = red; Before = continuous lines; after rSTS = dashed lines. Shaded rectangle = beta-band frequency (15 to 35hz).


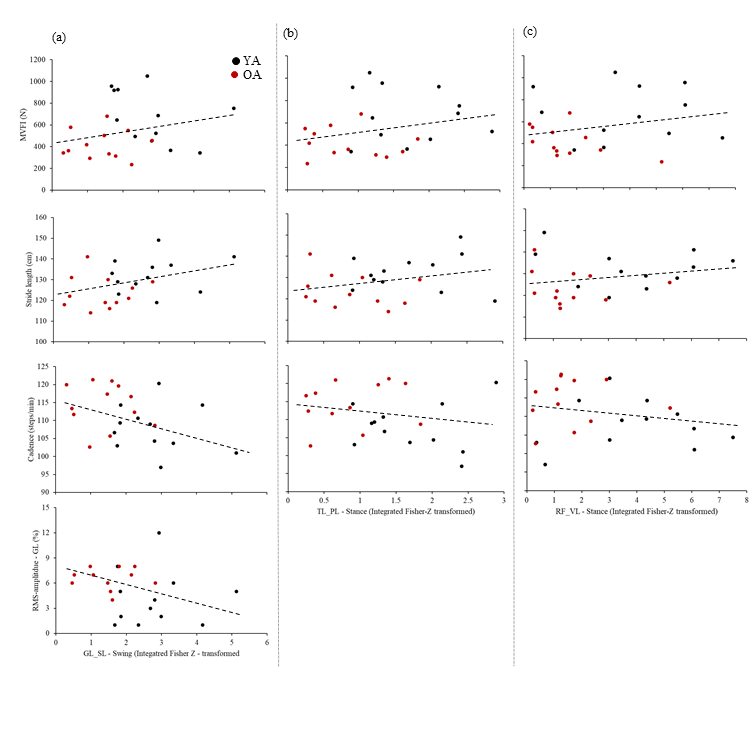


**Supplementary Figure 3.** Distributions plots of the association between beta-band coherence in GL-RF in swing (a), and TA-PL (b) and RF-VL (c) in stance with maximum voluntary isometric force (MVFI), stride length and cadence, outcomes that indicated age differences before rSTS.


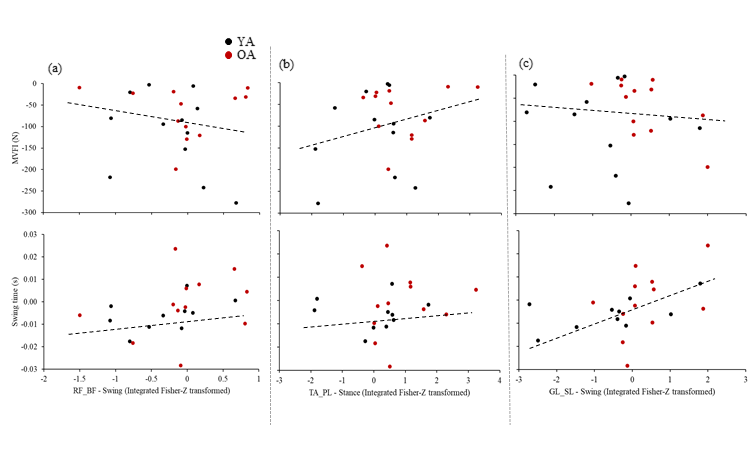


**Supplementary Figure 4.** Distributions plots of the association between absolute changes (after – before rSTS) in beta-band coherence in RF-BF (a) and TA-PL in stance (b), and GL-SL (c) in swing with maximum voluntary isometric force (MVFI) and swing time.
